# Mutualistic Co-evolution of Type III Effector Genes in *Sinorhizobium fredii* and *Bradyrhizobium japonicum*

**DOI:** 10.1371/journal.ppat.1003204

**Published:** 2013-02-28

**Authors:** Jeffrey A. Kimbrel, William J. Thomas, Yuan Jiang, Allison L. Creason, Caitlin A. Thireault, Joel L. Sachs, Jeff H. Chang

**Affiliations:** 1 Department of Botany and Plant Pathology, Oregon State University, Corvallis, Oregon, United States of America; 2 Molecular and Cellular Biology Program, Oregon State University, Corvallis, Oregon, United States of America; 3 Department of Statistics, Oregon State University, Corvallis, Oregon, United States of America; 4 Center for Genome Research and Biocomputing, Oregon State University, Corvallis, Oregon, United States of America; 5 Department of Biology, University of California-Riverside, Riverside, California, United States of America; University of Toronto, Canada

## Abstract

Two diametric paradigms have been proposed to model the molecular co-evolution of microbial mutualists and their eukaryotic hosts. In one, mutualist and host exhibit an antagonistic arms race and each partner evolves rapidly to maximize their own fitness from the interaction at potential expense of the other. In the opposing model, conflicts between mutualist and host are largely resolved and the interaction is characterized by evolutionary stasis. We tested these opposing frameworks in two lineages of mutualistic rhizobia, *Sinorhizobium fredii* and *Bradyrhizobium japonicum*. To examine genes demonstrably important for host-interactions we coupled the mining of genome sequences to a comprehensive functional screen for type III effector genes, which are necessary for many Gram-negative pathogens to infect their hosts. We demonstrate that the rhizobial type III effector genes exhibit a surprisingly high degree of conservation in content and sequence that is in contrast to those of a well characterized plant pathogenic species. This type III effector gene conservation is particularly striking in the context of the relatively high genome-wide diversity of rhizobia. The evolution of rhizobial type III effectors is inconsistent with the molecular arms race paradigm. Instead, our results reveal that these loci are relatively static in rhizobial lineages and suggest that fitness conflicts between rhizobia mutualists and their host plants have been largely resolved.

## Author Summary

Rhizobia are an important group of bacteria that can enter into mutually beneficial symbiotic interactions with legume plants to fix atmospheric nitrogen. However, in order to do so, a complex dialog involving the exchange of chemical and molecular signals must occur between partners. Some species of beneficial rhizobia employ a type III secretion system, a well-characterized virulence mechanism used by pathogens to inject bacterial-encoded type III effector proteins directly into host cells to coerce the host into accommodating the microbe. In this study, we generated draft genome sequences and employed computational as well as experimental methods to identify type III effectors from eight strains representing *Sinorhizobium fredii* and *Bradyrhizobium japonicum*. We demonstrate that the type III effector genes of these rhizobial species are highly conserved in content with little diversity between strains. This work is an important step towards understanding the roles for type III secretion systems and their effectors in mutualistic interactions.

## Introduction

Eukaryotes universally encounter bacteria that inhabit, infect, and often provide them with significant fitness benefits. In many cases, bacterial mutualist lineages exhibit intimate interactions with hosts, giving each partner opportunity to shape the phenotype of the other. Two diametric paradigms remain unresolved for the co-evolution of bacterial mutualists with their eukaryotic hosts [1]. One common paradigm models mutualist-host interactions as an antagonistic arms race, as is the case for co-evolution of pathogens and their hosts. Under this model, natural selection is predicted to shape partners to rapidly evolve traits to maximize their own selfish gains from the interaction and minimize costs invoked by the other [1]. This paradigm predicts that there is constant conflict over the fitness gain that each partner receives from the interaction even though both partners can attain net fitness benefits. The alternative framework assumes that conflicts between microbe and host are largely resolved [2], [3]. It is predicted that the common genotypes are more likely to find compatible partners than the rare genotypes. As a consequence, the interaction is predicted to exhibit evolutionary stability, with lower rates of evolutionary change. Testing these competing frameworks by comparing the genetic patterns of known host-association genes between mutualists and pathogens will help to examine whether bacteria-eukaryotic mutualisms represent reciprocally exploitative interactions, as they have often been characterized, or alternatively, if these interactions exhibit a “mutualistic environment” in which evolutionary stasis is maintained [1], [3].

A striking and well-studied example of arms race co-evolution occurs between proteobacterial pathogens and plant hosts. Plants have multiple defense systems to recognize and respond to bacterial infection. One key plant defense is pattern-triggered immunity (aka PAMP-triggered immunity; PTI), in which pattern recognition receptors detect conserved microbe-associated molecular patterns and trigger defenses [4]. To counteract host defenses, many phytopathogenic bacteria use type III secretion systems (T3SS) to deliver collections of type III effector proteins (T3Es) to dampen host defenses, thereby allowing the bacteria to proliferate within host tissues and cause disease. A second line of host defense is effector-triggered immunity (ETI) in which resistance (R) proteins surveil for corresponding microbial effectors to trigger a robust defense often associated with a localized programmed cell death (hypersensitive response; HR).

Plant pathogen T3Es exhibit patterns of genetic variation that reflect rapid evolution, as predicted by the antagonistic arms race model [1], [5]–[8]. In *Pseudomonas syringae*, the phytopathogenic species with the most extensive experimentally-validated set of T3Es, strains vary dramatically in T3E gene content, both in terms of the total number and sequence of effector genes [1], [7]. Even highly related strains exhibit T3E presence/absence polymorphisms and insertion/deletion mutations that affect their coding sequences [2], [3], [9]. An important aspect of pathogen T3E collections is that their robustness is ensured via T3E redundancy so that any individual T3E gene is dispensable [1], [3], [10]. Hence, under the arms race scenario, rapid evolution of T3Es is advantageous to phytopathogens as novel collections of T3Es are more likely to avoid recognition while balancing sufficiency in subverting host defenses.

Functional T3SS orthologs have been uncovered in diverse mutualistic species of proteobacteria, including nitrogen-fixing rhizobial species *Sinorhizobium fredii* (*Ensifer fredii*), *Bradyrhizobium japonicum*, and *Mesorhizobium loti* [4], [11], [12]. Analyses of T3SS and T3E (Nops; Nodulation Outer Proteins) of rhizobia reveal many parallels to those of phytopathogens, pointing to the possibility that rhizobial *nop* genes are also under selection to maximize rhizobial fitness at potential expense of the fitness of the host. For instance, multiple studies have shown that T3SS and Nops of rhizobia are necessary for the establishment of mutualist infections and can modulate host PTI [13]–[20]. Moreover, T3Es of rhizobia also risk detection by host defense surveillance systems. In fact, legume loci responsible for “nodulation restriction” are *R* genes that restrict rhizobia in a T3SS-dependent manner and are linked to loci associated with resistance against phytopathogens [21]–[24]. This is consistent with the repeated observations that rhizobial strains deleted of genes encoding T3SS-secreted proteins gain new hosts that were once incompatible [19], [24], [25]. Since no study has examined the molecular evolution of T3Es in the context of mutualism, it is presently unknown whether these lineages exhibit patterns of genetic variation that would reflect arms race evolution with their hosts [5]–[8].

To this end, we investigated the molecular evolution of T3E genes in two lineages of mutualistic rhizobia and tested the arms race versus mutualistic environment paradigms. We used an experimentally validated set and compared their genetic patterns against the patterns of T3Es from five monophyletic strains of *P. syringae* (group I strains) and four that infect legumes (legume pathovars) to test the null hypothesis that collections of T3Es of mutualists evolve in a manner similar to those in proteobacterial phytopathogens.

## Results/Discussion

### Draft genome sequencing and genetic diversity of *S. fredii* and *B. japonicum*

We selected three *S. fredii* and five *B. japonicum* strains based on the criterion of demonstrable reliance on T3SS for host infection [11]. For *B. japonicum*, we also chose strains based on the genetic diversity inferred from their phylogenetic relationship [26]. At the initiation of this study, the only available finished genome sequences were for *S. fredii* NGR234 and *B. japonicum* USDA110 [27], [28]. We used paired-end Illumina sequencing to generate draft genome sequences for *S. fredii* USDA207, USDA257, and *B. japonicum* USDA6, USDA122, USDA123, and USDA124 (Table S1). Initial and *post hoc* analyses based on comparisons to reference and corresponding finished genome sequences completed subsequent to our efforts, respectively, indicated that the assemblies and annotations are of sufficient quality and covered the majority of the genomes for whole-genome characterization and comprehensive genome mining (Figure S1).

**Figure 1:**
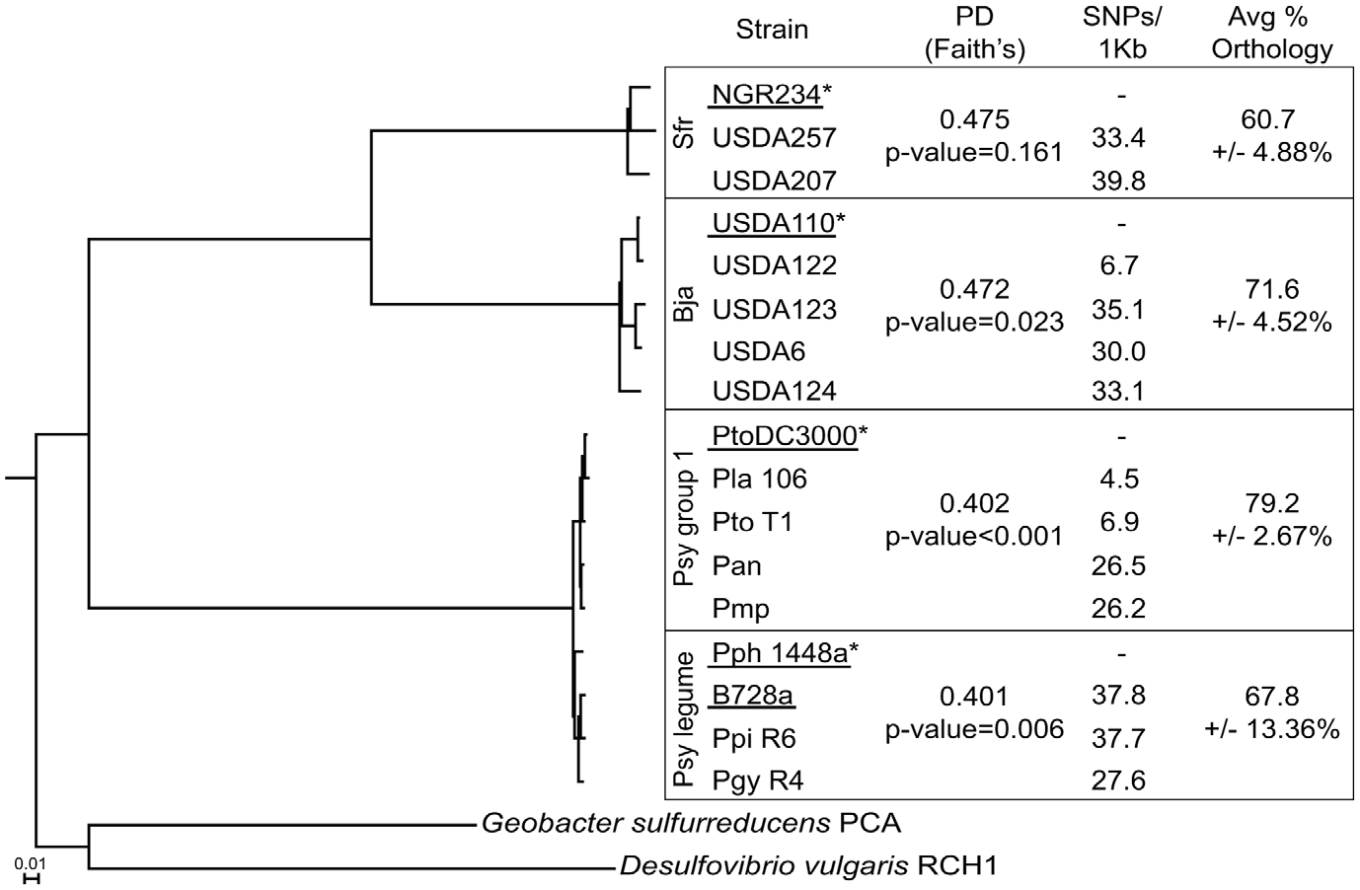
Within-group genetic diversity for *S. fredii*, *B. japonicum*, is higher than the diversity within the *P. syringae* groups. A rooted tree was constructed from the concatenated sequences of 103 genes present in all 17 strains and *Geobacter sulfurreducens* PCA and *Desulfovibrio vulgaris* used as outgroups. The scale bar indicates the number of amino acid substitutions per site. Phylogenetic divergence (PD) was measured for each group and compared to randomly assigned groups of strains. Reliable SNPs, based on pairwise comparisons to group-specific reference strains (*), were identified and calculated per kb (see Figure S1). The percent orthology was averaged from all within-group pairwise comparisons (see Figure S2). Each group included strains with finished (underlined) and draft genome sequences.

We next used multiple measures to compare the within-group diversity for the rhizobial groups to that of the group I and legume *P. syringae* pathovars to determine the suitability of the latter two for genomic comparisons (Figure 1; [7]). Quantitative measures of phylogenetic diversity (PD) fell within a narrow range with the two rhizobial groups having the higher PD values [29]. We also compared bacterial group PD values to those derived from equally sized groups of strains randomly assigned from the 17 used in this study. The within-group diversity of *S. fredii*, *B. japonicum*, and *P. syringae*, are similar, marginally, and significantly lower, respectively, relative to expectations due to chance. Additional measures based on average reliable single nucleotide polymorphisms (SNPs) per kilobase (kb) and average percent of orthologous pairs of genes were also consistent (Figures 1, S1, and S2). In total, the data demonstrate that the levels of genome-wide, within-group genetic diversity are higher in the *S. fredii* and *B. japonicum* groups, respectively, relative to either of the *P. syringae* groups.

### Genome mining for candidate type III effector genes

Candidate T3E genes were identified based on their association with a *tts*-box, a *cis* element proposed to be recognized by TtsI, a regulator of T3SS genes in rhizobia [13], [16]. We identified a total of 305 putative *tts-*boxes (Table 1). In *S. fredii* NGR234, we identified two additional *tts*-box sequences that were not previously reported [13]. In the finished genome sequence of *B. japonicum* USDA110, we found 52 *tts-*boxes, of which 29 were previously identified (Table 1; [30]). Fourteen of these *tts*-boxes are located upstream of 13 genes (*bll1862* has two upstream *tts-*boxes) that encode proteins that are secreted in a T3SS-dependent manner [30]. We searched up to 10 kb downstream of the 305 *tts*-boxes and identified a total of 268 candidate T3E genes that clustered into 92 different families (Table 1).

**Table 1:** Statistics for genome mining for T3E-encoding genes.

Strain	# *tts*-boxes*	# candidate T3Es†	# confirmed T3Es‡
*S. fredii*			
NGR234	13	24	15
USDA207	21	19	13
USDA257	24	21	13
*B. japonicum*			
USDA6	46	39	33
USDA110	52	49	36
USDA122	50	39	31
USDA123	47	37	32
USDA124	52	40	33

* A trained Hidden Markov Model (HMM) was used to identify candidate *tts*-boxes;

† CDSs within 10 kb and encoded on the same strand as the predicted *tts*-box were identified;

‡ T3E-encoding genes based on T3SS-dependent elicitation of HR by *Pt*oDC3000 in Arabidopsis Col-0.

### Functional testing of type III effectors for T3SS-dependent translocation

We adopted the Δ79AvrRpt2 reporter in the γ-proteobacterium *P. syringae* pv. *tomato* DC3000 (*Pto*DC3000) for high throughput testing of candidate rhizobial T3E for T3SS-dependent translocation into plant cells, the most important criterion for defining a T3E [31]. We first selected NopB and NopJ from *S. fredii* NGR234 as likely T3E candidates for validation of heterologous T3SS-dependent translocation. NopB is secreted *in vitro* in a flavonoid- and T3SS-dependent manner from *S. fredii* NGR234, and NopJ is a member of the YopJ/HopZ T3E family [32], [33].

**Figure 2:**
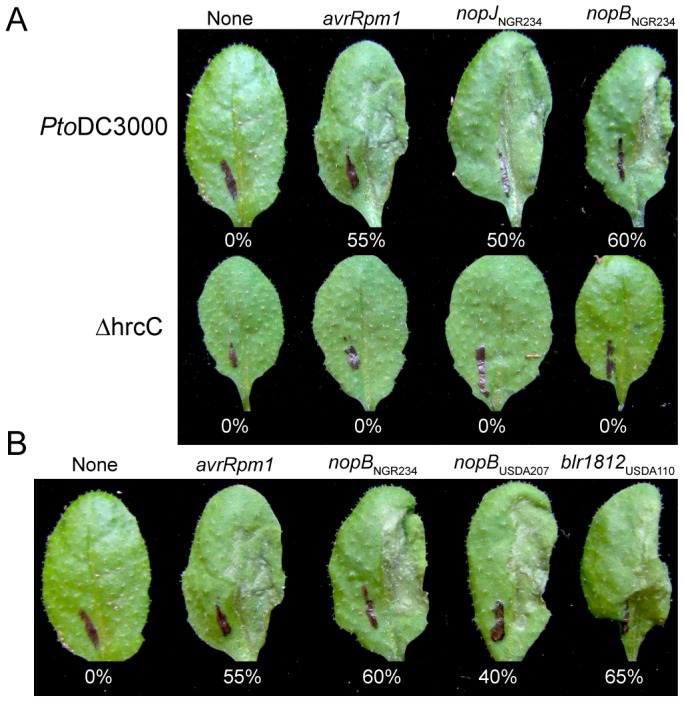
*Pto*DC3000 delivers T3Es of rhizobia in a T3SS-dependent manner. (A) Leaves of Arabidopsis Col-0 (*Rps2*/*Rps2*) were infiltrated with *Pto*DC3000 (top row) and its T3SS-deficient mutant, Δ*hrcC* (bottom row) carrying no fusion to Δ79*avrRpt2* or fusions to *P. syringae* T3E *avrRpm1* or coding sequences from NGR234 candidate T3E genes, *nopJ* or *nopB*. (B) Members of the NopB T3E gene family all encode for functional T3Es. Leaves of Arabidopsis Col-0 (*Rps2*/*Rps2*) were infiltrated with *Pto*DC3000 carrying no fusion to Δ79*avrRpt2* or fusions to *P. syringae* T3E *avrRpm1* or *nopB* coding sequences from NGR234, USDA207, or USDA110. Leaves did not respond to infiltrations of Δ*hrcC*. In all experiments, leaves were scored for the HR ∼20 hpi and the percent of responding leaves are presented (at least 20 leaves infiltrated). Experiments were repeated at least three times.

*Pto*DC3000 carrying either the *nopB*::Δ*79avrRpt2* or *nopJ*::Δ*79avrRpt2* fusions elicited HRs within the same time frame (∼20 hours post inoculation; hpi) and to the same degree as the positive control, a fusion between the full-length *avrRpm1 P. syringae* T3E gene and Δ*79avrRpt2* (Figure 2A). Although Arabidopsis ecotype Col-0 can elicit ETI in response to both AvrRpm1 and AvrRpt2, the observed HR is known to be a consequence of perception of the latter by RPS2 [34]. Each of the tested *nopB*::Δ*79avrRpt2* gene fusions were sufficient for *Pto*DC3000 to trigger an HR at 20hpi, confirming that this family encodes *bona fide* T3Es (Figure 2B). The NopB family is polymorphic with NopB_NGR234_ sharing ≥98% amino acid identity with NopB_USDA207_, but only 32% with NopB_USDA110_. In contrast, *Pto*DC3000 lacking fusions to Δ*79avrRpt2* failed to elicit an HR but eventually showed tissue collapse approximately 28 hpi, indicative of *Pto*DC3000-caused disease symptoms (data not shown). The T3SS-deficient mutant of *Pto*DC3000 (Δ*hrcC*), regardless of the gene it carried, failed to elicit any phenotype throughout the course of the study, thereby demonstrating the T3SS-dependent delivery of T3Es (Figure 2A).

**Figure 3:**
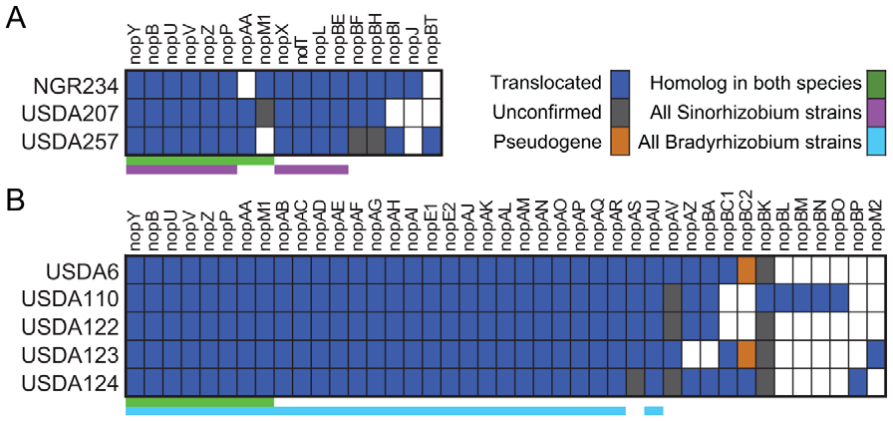
Distribution of T3E families in rhizobia. The T3E family names are listed across the top with strains of (A) *S. fredii* and (B) *B. japonicum* listed down the side. Boxes are color-coded as indicated in the key; white boxes = no detectable homolog. Conservation of T3Es is also color-coded (bars below each chart) as indicated.

The demonstration that members of a polymorphic T3E family behaved identically in the heterologous delivery assay allowed us to test just a subset of 127 genes that represent the diversity present in the 268 candidates. From these, 87 T3Es belonging to 47 families between the two rhizobial lineages were confirmed for T3SS-dependent translocation (Table 1; Figure 3). We also used the sequences of members of confirmed T3E families to re-survey all draft genome sequences and identified an additional 21 homologs that were interrupted by physical and sequence gaps. Nine CDSs were amplified using PCR and sequenced and all were classified as functional based on the absence of premature termination codons. The remaining 12 genes belonged to 10 families with four homologs having upstream sequences similar to a *tts-*box but with bit-scores below our threshold. Seven had no discernible upstream *tts*-box, and one (*nopM2*) potentially represents a subgroup of the *nopM* family since two copies are present in *B. japonicum* USDA123.

Of the 24 candidate and confirmed T3E families that were identified prior to this study, our computational method identified 21 of which 19 were experimentally validated as T3Es (Table S2; [35], [36]). Of the five that we failed to confirm, NopA may in fact be a secreted structural component of the T3SS [37]. NopT, in contrast, is likely a *bona fide* T3E but its cytotoxic effects in Arabidopsis could have caused misleading conclusions in the translocation assay [18]. NopC, NopH and NopD lacked a detectable *tts*-box or failed to meet the requirement of being >100 amino acids in length.

The T3Es were assigned to families according to guidelines developed for T3Es of pathogenic bacteria [38]. Newly identified T3E families were assigned NopY through NopBT whereas 16 previously named families, that were confirmed in this study as representing T3Es, remain unchanged [11]. A relational table of the validated T3E genes is provided (Table S2). Other than those previously identified, none of the translated sequences of the T3E genes identified in this study have detectable homology to proteins of known function.

### Type III effector collections of *S. fredii* and *B. japonicum* are conserved

We compared the genetic patterns of the rhizobial T3Es to: 1) those of the group I strains of pathogenic *P. syringae*, and 2) the core genome of the respective bacterial groups (i.e., genes ubiquitous to all strains within a group) to test the null hypothesis that rhizobial T3Es exhibit signatures of arms race evolution similar to what has been characterized in pathogenic *P. syringae* lineages [5]–[8].

**Figure 4:**
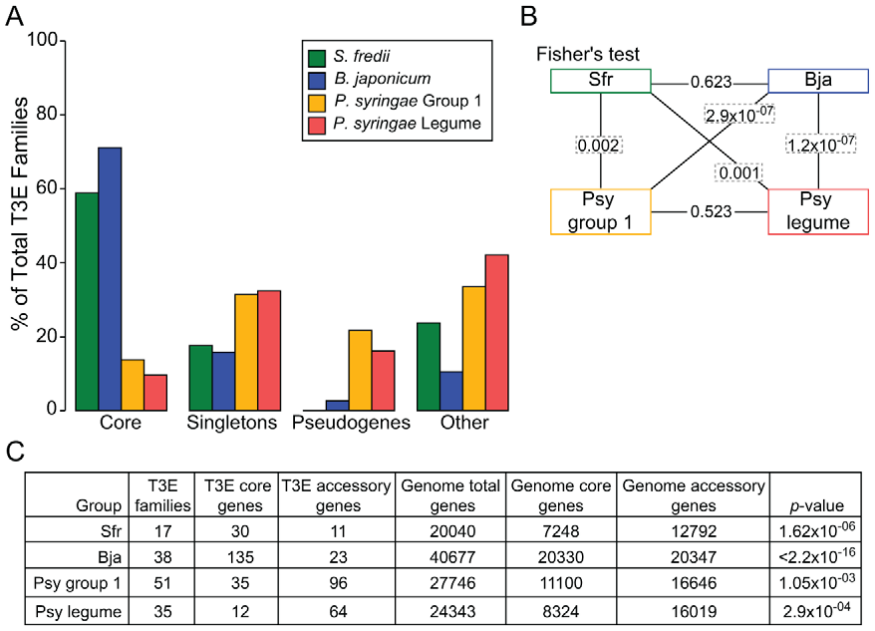
T3E collections of *S. fredii* and *B. japonicum* are highly conserved in content. (A) Representation of T3Es in categories as percentage of total number of T3E families in *S. fredii*, *B. japonicum*, as well as group I and legume pathovars of *P. syringae*. Unconfirmed T3Es were not included. (B) Fisher's exact test for all pairwise comparisons (connected by lines) of the representation of T3Es in the four categories depicted in panel (A). Boxed *p*-values are significant (Bonferonni adjusted α level = 0.0083). (C) Numbers of T3E genes and all genes binned as total, core, or accessory for each group of bacteria. Core genes are defined as those with orthologs present in all strains within each group. A Fisher's exact test was used to test for differences in distribution of core and accessory T3E genes relative to the distribution of all genes. All *p*-values are significant (Bonferonni adjusted α level = 0.0125).

The T3Es of rhizobia were predominantly core, unlike the T3Es of the group I strains of *P. syringae* (Figure 4A). In fact, the representation of T3Es among the four categories of core, singletons (present in only a single strain of a group), pseudogenes (premature termination codon relative to a full-length family member), and other (polymorphic in regards to presence/absence), was significantly different (Figure 4B). Next, we compared the proportion of core and accessory T3E genes in the *S. fredii*, *B. japonicum*, and *P. syringae* group I strains to the proportion of genes that are core and accessory to each group (Figure 4C). Analysis indicated that the proportions of core T3E genes were significantly more than core genes for both groups of rhizobia. In contrast, the proportion of core T3E genes for the group I strains of *P. syringae* was significantly less. Thus, the collections of T3E genes of rhizobia are significantly more conserved than the collection of T3E genes of *P. syringae* and relative to their core genomes.

**Figure 5:**
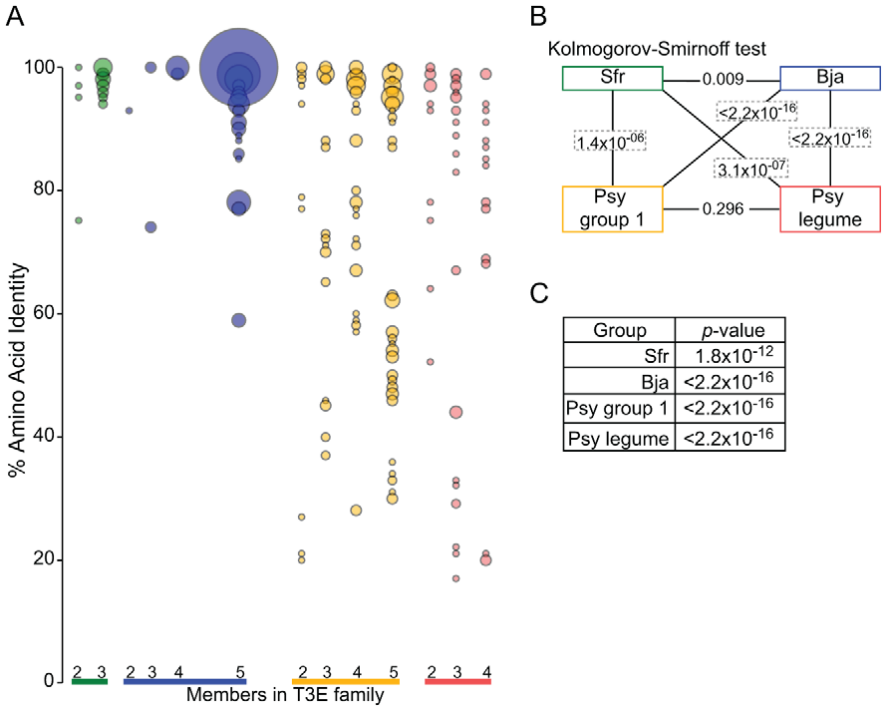
T3E of *S. fredii* and *B. japonicum* have high levels of within-family amino acid identity. (A) Balloon plots of within-family amino acid conservation for translated T3Es. The percent amino acid identity was calculated for all pairwise comparisons within each family (y-axis) and plotted according to the number of members within families (x-axis). The sizes of the balloons are scaled with the largest representing 162 pairwise comparisons (the smallest balloons were enlarged). Unconfirmed T3E and pseudogene sequences were not included in the comparisons. (B) Kolmogorov–Smirnov test for all pairwise comparisons (connected by lines) of the distributions depicted in panel (A). Boxed *p*-values are significant (Bonferonni adjusted α level = 0.0083). (C) An F test for linear hypothesis was used to test for differences in percent amino acid identity for translated T3Es and core genes within each group. All *p*-values are significant (Bonferonni adjusted α level = 0.0125).

The sequences within T3E families of *S. fredii* and *B. japonicum* are also highly conserved, as more than 75% of the within-family pairwise comparisons had ≥90% amino acid identity (Figure 5A). Strikingly, twenty of the T3E families had all members with ≥99% identity. The T3Es of the group I *P. syringae* strains have a wider distribution in amino acid identity and a greater number of presence/absence polymorphisms than *S. fredii* or *B. japonicum*. Even when the latter variation was excluded from analysis, *S. fredii* and *B. japonicum* exhibit significantly more amino acid conservation of T3Es than group I *P. syringae*, whereas there was only marginal difference between the rhizobial lineages (Figure 5B).

To determine whether the levels of sequence conservation of T3E gene families differed relative to genes core to their respective genomes, we calculated and compared the within-family amino acid identity for the translated sequences of gene families core to each of the groups (Figure 5C). The T3E gene families were significantly more conserved in sequence in both groups of rhizobia. In contrast, for the five group I *P. syringae* strains the translated sequences of the T3Es exhibited significantly lower amino acid identities as compared to the translated sequences of the core gene families. Therefore, relative to their respective core genes, the T3E genes of rhizobial and *P. syringae* lineages differed, with the former displaying higher levels of sequence conservation and the latter having significantly lower conservation.

### Variation in T3E conservation is not explained by variation in host range

There is no clear relationship between T3Es and host range of pathogens [7], [39]. *P. syringae* strains that infect the same host possess substantially different collections of T3Es. For example, *P. syringae* pairs, pvs. *tomato* races DC3000 and T1 and *lachrymans* races 106 and 107, share no more than 50% of their T3Es in common [7], [40]. The high variability in T3Es is in spite of the lower levels of genetic diversity detected relative to most pairs of rhizobial strains (Figures 1 and S2; [9]). It has been suggested that *Xanthomonas* pathovars with similar hosts share similar compositions of T3Es [41], [42]. However, the genome-wide diversity is unknown for these bacteria and furthermore, use of contemporary methods to study two *Xanthomonas* species has revealed a surprisingly high number of pseudogenized T3Es and divergence in T3E collections [43], [44].

For *P. syringae*, it is hypothesized that T3Es are capable of functioning in a range of plant species [39]. The extensive host ranges for two of the rhizobial strains studied herein support this notion. *S. fredii* NGR234 and USDA257 can infect 112 and 79 genera of host plants, respectively, many of which are not considered cultivated plants and are more apt to have high within-population genetic diversity [45]. Support is further bolstered by the observation that *P. syringae* deleted of a T3E gene gains the ability to infect an otherwise non-host plant [46]. Similarly, rhizobial mutants deficient in secretion of T3SS-associated proteins can gain new species of plants as hosts [19], [24], [25].

To further test the potential for host range as a factor in the conservation of rhizobial T3Es, we compared their genetic patterns to those of T3Es from four *P. syringae* pathovars that like rhizobia, can infect legumes as hosts [7]. First, we compared between the two *P. syringae* groups. As expected, the within-group genetic diversity is similar (Figures 1 and S2). The genetic patterns of T3Es of the legume pathovars do not deviate significantly from those of the group I strains (Figures 1, 4, and S2). Finally, relative to core genes, the core T3Es of the legume pathovars have genetic patterns that are significantly different (Figure 4C and 5C). Thus, despite the fact that the legume pathovars are distributed between two *P. syringae* groups, they exhibit similar levels of genome-wide and T3E diversity as the group I strains.

Relative to the T3Es of *S. fredii* and *B. japonicum*, the T3Es of the legume pathovars are significantly more variable. As was the case for comparisons to the group I strains, the representation of core, singletons, pseudogenes, and other T3E categories is significantly different between legume mutualist and legume pathovars (Figure 4A and 4B). Likewise, the T3Es of the legume mutualists have a significantly different distribution in percent amino acid identity within T3E families relative to those of the legume pathovars (Figures 5A and 5B). Therefore, we conclude that a difference in host range is not a likely explanation for the extreme contrasts in T3E conservation between mutualist and pathogen.

### Conclusions

The type III secretion system is a key mechanism used by a diversity of bacterial mutualists to establish infections with their hosts. We identified and validated type III effectors to test the two diametric frameworks of mutualist-host co-evolution (Figures 2 and 3; Table S2). Rhizobial T3E genes show genetic patterns indicative of surprising conservation, pointedly contrasting the patterns consistent with the dynamic arms race model of co-evolution dogmatic for T3Es (Figures 3–5). This finding is particularly striking in light of the observations that T3Es of mutualistic rhizobia are similar in regards to those of pathogens in having to maintain sufficiency in engaging and dampening PTI while avoiding ETI [15], [17], [24], [47]. Moreover, we demonstrated that the high conservation of T3Es in rhizobia relative to phytopathogens is not likely driven by differences in host range or phylogenetic diversity among genomes (Figures 1, S2, 4, and 5). The high conservation in sequence and the fact that most of the T3E loci are co-localized are also consistent with acquisition events by both species of rhizobia. In *B. japonicum*, for example, most of the T3E genes are found distributed throughout an ∼700 kb-long symbiosis island. However, analysis of *B. japonicum* USDA110 and USDA6 suggested that the symbiosis islands were acquired independently, arguing against a common genome innovation event [48].

We favor an alternative explanation that the relative conservation of rhizobial T3Es reflects the selective pressures in these beneficial plant-microbe interactions. It has been suggested that legume hosts exhibit less polymorphisms in loci that restrict nodulation, in contrast to the higher levels of polymorphisms observed in loci that mediate resistance against phytopathogens [49]. Our data support this idea that novelty in mutualism can result in instability, specifically that rhizobial mutualists may be under pressure by the host that limits diversification [50], [51]. In this context, hosts select for the most beneficial rhizobial genotype and these consequently common genotypes are more likely to find a suitable host. The type III effectors of *S. fredii* and *B. japonicum* thus exhibit mutualistic co-evolution with host defenses.

## Materials and Methods

### Bacterial strains and growth conditions

Bacterial strains used in this study were: *S. fredii* strains USDA207 and USDA257; *S. fredii* (aka *Rhizobium* sp.) NGR234; *B. japonicum* strains USDA6, USDA110, USDA122, USDA123, and USDA124; *Pto*DC3000, its T3SS-deficient mutant (Δ*hrcC*), and *Escherichia coli* DH5α. Rhizobia strains and *P. syringae* were grown in modified arabinose gluconate media (MAG) or King's B (KB) media, respectively, at 28°C. *E. coli* DH5α was grown in Luria-Bertani (LB) media at 37°C. Antibiotics were used at the following concentrations: 50 µg/ml rifampicin (*Pto*DC3000), 30 µg/ml kanamycin (all bacterial strains), 50 µg/ml chloramphenicol (*B. japonicum* strains), and 25 µg/ml gentamycin (*E. coli*).

### Genome sequencing and bioinformatics

Genomic DNA was extracted from *S. fredii* strains USDA207 and USDA257 and *B. japonicum* strains USDA6, USDA122, USDA123, and USDA124 using osmotic shock, followed by alkaline lysis and phenol-chloroform extraction. We prepared 5 µg of DNA from each strain according to the instructions provided by the manufacturer (Illumina, San Diego, CA). Paired-end sequencing was done by the Center for Genome Research and Biocomputing Core Labs (CGRB; Oregon State University, Corvallis, OR; Table S1). Velvet 0.7.55 was used to *de novo* assemble paired-end short reads [52]. Multiple assemblies, using different parameters, were produced for each genome and the highest quality assembly was identified using methods described previously [53]. Genomes were annotated using Xbase and further refined using the NCBI conserved domain database (CDD; [54]–[61]). The Mauve Aligner 2.3 (default settings) program was used to compare the draft and finished genomes and, in other instances, reorder contigs to reference sequences [62].

To identify SNPs, we used Bowtie ver. 0.12.5 to align short reads to the finished genome sequence, allowing up to two mismatches [63]. Reliable sequence differences were identified based on having coverage of ≥10 reads and ≥8 reads supporting the same alternative base call. For *P. syringae*, we treated the publicly available genome sequences as true and incremented along the genome in 1 base pair increments, shearing *in silico* the genome into 32mers, and aligned the sequences to the indicated reference genome sequence.

Homologous sequences were identified using reciprocal BLASTP analysis (e-value≤1×10^−15^; >50% length of sequence) of translated sequences (those <50 amino acids in length were excluded). The Circos plot was generated using the Circos Table Viewer [64].

Genome sequences were retrieved from http://www.ncbi.nlm.nih.gov/genome: *S. fredii* NGR234 (NC_012587), *S. fredii* USDA257 (NC_018000), *B. japonicum* USDA6T (NC_017249), *B. japonicum* USDA110 (NC_004463), the *P. syringae* pathovars, *actinidiae* (Pan; AEAL00000000), *glycine* (Pgy R4; ADWY00000000), *lachrymans* (Pla 106; AEAM00000000), *morsprunorum* (Pmp; AEAE00000000), *phaseolicola* (Pph 1448a; NC_005773), *pisi* (Ppi R6; AEAI00000000), *syringae* (B728a; NC_007005), *tomato* (*Pto*DC3000; NC_004578), and *tomato* (Pto T1; ABSM00000000). Finished genome sequences from *S. fredii* USDA257 and *B. japonicum* USDA6T were used for *post hoc* analysis of genome assemblies [48], [65].

We used HAL (default settings) to identify clusters of orthologous genes and generate a whole-genome phylogeny of the 17 strains plus two δ-proteobacterial reference strains, *Geobacter sulfurreducens* PCA (NC_002939) and *Desulfovibrio vulgaris* RCH1 (NC_017310), used as outgroups [66]. PD values were calculated using the Picante R package [67].

### Statistical analyses

To calculate PD values for randomly assigned groups of three, four, five, and five strains, an *ad hoc* Perl script was used to randomly assign the 17 rhizobial and *P. syringae* strains into four groups. The process was iterated 1000 times and PD values were calculated for each group per iteration. Statistical significance was determined by comparing the observed PD values to the proportion of 1000 iterations that had higher or lower PD values than the observed PD values.

To identify the proportion of core genes for each group of strains, we identified the clusters of orthologous genes, generated by HAL, that were represented by all strains within each group. Fisher's exact test was used to compare the representations of T3E genes in the four categories for all possible pairs of bacterial groups [68]. The Kolmogorov-Smirnov test was used to compare the distributions of percent amino acid identity of T3E genes for all pairwise comparisons [69].

We developed a linear regression model that evaluates the average percent amino acid identity for both core and T3E families, using the core genes in the group I strains of *P. syringae* as the baseline:
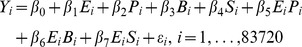
where *Y* = the response variable, percent amino acid identity; *P* = 1 for the legume pathovars of *P. syringae* and *P* = 0 otherwise; *B* = 1 for the *B. japonicum* species and *B* = 0 otherwise; *S* = 1 for the *S. fredii* species and *S* = 0 otherwise; *E* = 1 for T3E families and *E* = 0 for core gene families; *ε* = random error.

Specifically, *ß_0_* measures the average percent amino acid identity of the core genes in the group I strains of *P. syringae*; *ß_1_* measures the difference in percent amino acid identity between the T3E families and the core gene families for the group I strains of *P. syringae*; *ß_2_*, *ß_3_* and *ß_4_* measure the differences in percent amino acid identity for the core gene families for the legume pathovars of *P. syringae*, *B. japonicum*, and *S. fredii*, respectively, against that of the group I strains; *ß_5_*, *ß_6_* and *ß_7_* allow the variation of the differences in percent amino acid identity between T3Es and core gene families across the groups; in particular, *ß_1_+ß_5_*, *ß_1_+ß_6_* and *ß_1_+ß_7_* measure the differences in percent amino acid identity between the T3E families and the core gene families for the legume pathovars, *B. japonicum*, and *S. fredii*, respectively.

An F test was used to test the null hypotheses that the percent amino acid identity for within-family comparisons between translated T3E and core gene sequences are equal within bacterial groups: *ß*_1_ = 0, *ß*_1_+*ß*_5_ = 0, *ß*_1_+*ß*_6_ = 0 and *ß*_1_+*ß*_7_ = 0 (F test with degrees of freedom 1 and 83712).

A Bonferroni correction was used when applicable [70].

### T3E candidate discovery, cloning, and testing

We used sequences of 30 confirmed functional *tts*-boxes from *B. japonicum*, *S. fredii* and *M. loti* MAFF303099 to train a Hidden Markov Model [13], [30], [71]. To identify candidate T3E genes, we identified CDSs downstream of *tts*-boxes with bit scores ≥5.0, calibrated based on the identification of 11 functionally validated *tts*-boxes located on the pNGR234a plasmid [13]. To be considered, CDSs had to be encoded on the same strand as the *tts-*box, either up to 10 kb downstream or until another CDS on the opposite strand was encountered. TtsI-regulated operons, such as the *nopB*-*rhcU* operon of *S. fredii* NGR234, can be substantial in length [72]. We used BLASTX (e-value≤1×10^−15^) to filter out CDSs with translated sequences homologous to components of the T3SS, proteins encoded by organisms that lack a T3SS, or proteins with general housekeeping functions. We used BLASTN and sequences of candidate T3E-encoding genes to identify homologs from each of the eight genome sequences (e-value cutoff≤1×10^−15^).

T3Es were grouped into families based on BLASTP scores ≤1×10^−5^ across ≥60% the length of the protein [38]. When all members of a family had amino acid identity ≥90% as determined using ClustalW, a single representative family member was chosen for testing [73]. In families of <90% amino acid identity, members representative of the diversity were tested. PCR, Gateway cloning into pDONR207 and the destination vector pDD62-Δ79AvrRpt2, transformation into *E. coli* DH5α cells, and triparental mating into *Pto*DC3000 or Δ*hrcC* were done as previously described or according to the instructions of the manufacturer (Invitrogen, Carlsbad, CA; [31]). Infiltration and HR assays were done as previously described [31]. Plants were grown in a controlled growth chamber environment (15-hour day at 22°C followed by 9-hour night at 20°C). Experiments were replicated a minimum of three times.

## Supporting Information

Figure S1 **Quality assessment of representative draft genome assemblies and SNP calls.** (A) Alignments of 384 and 788 contigs from *S. fredii* USDA257 and (B) *B. japonicum* USDA6, respectively, to their corresponding genome sequence (NC_018000 and USDA6T, respectively) that were finished subsequent to our efforts. The conservation in order of locally collinear blocks (LCBs) and the heights of the colors can be used to infer quality of assembly. A visual inspection of LCBs identified only a few small regions that are potentially misassembled. Additionally, ∼81% and ∼96% of the coding sequences (CDSs) in the draft genome sequence were similarly annotated in the finished genome sequences, respectively. (C) Mapping of Illumina short reads from *B. japonicum* USDA6 to the finished genome sequence of *B. japonicum* USDA6. Black dots indicate the depth of coverage (y-axis) over a 10 kb interval slid along 1.0 kb increments, relative to genome position (x-axis). The 34 red dots (some are overlapping on this scale) approximate the positions of reliable sequence differences based on having ≥10 reads (indicated by the dotted line) and ≥8 reads supporting the same alternative base call. There are nine and six highly supported sequence differences located in the two 16S rRNA regions (blue bars) that are hypervariable and detected as polymorphisms because of the misalignment of reads ambiguous to the regions. There were only 19 other sequence differences identified from >9.0 Mb of sequence with >10× coverage that could be either true isolate-specific differences and/or sequencing errors, suggesting the method used to identify SNPs leads to few false positives.
(EPS)

Figure S2 ***S. fredii*** **,** ***B. japonicum*** **, and** ***P. syringae*** **have similar amounts of genome-wide orthology.** (A) Orthologous genes were determined for all pairwise, within-group comparisons for *S. fredii* (top left), *B. japonicum* (top right), and two *P. syringae* groups (bottom left = group I; bottom right = legume pathovars). The percent orthology was calculated relative to the number of genes (second row; gray boxes) annotated for the strains listed along the top. (B) Circos visualization of genome-wide orthology within and between *S. fredii* and *B. japonicum* strains. The most outer track, based on the length of the different colored bars, represents the amount of orthology other genomes have to the indicated genome. Genomes are ranked according to highest percent orthology (starting close to 0%) to lowest (ending closer to 100%). The inner track represents the amount of orthology the indicated genome has to the other genomes with interior ribbons connecting genomes, and variation in width depicting the extent of orthology. Genomes were assigned arbitrary colors (in a counter-clockwise direction: NGR234, dark blue; USDA207, blue; USDA257, cyan; USDA6, maroon; USDA110, olive; USDA122, gray; USDA123, orange; and USDA124, yellow).
(EPS)

Table S1 **Statistics for genome assemblies.** *Finished prior to initiation of this study; ^†^32mer and 72mer reads were generated on an Illumina IG or GAII, respectively; ^‡^number of usable paired end reads; ^§^greater than 100 nt in length; ^¶^estimates based on sum total of contigs; ^∥^contigs were ranked ordered according to size and the size (kb) of the smallest contig of those sufficient to represent 50% of the genome size is presented; ^**^number of predicted coding sequences greater than 150 nt in length.
(PDF)

Table S2 **Nop relational table.** *Nop = Nodulation outer protein; Nop designations determined according to guidelines proposed in (1–2). ^†^ *S. fredii* (NGR234, USDA207 & USDA257) and *B. japonicum* (USDA6, USDA110, USDA122, USDA123, & USDA124). ^‡^X = tested; − = not tested but was considered functional if it had >90% amino acid identity to a confirmed T3E identified from a tested strain. ^§^Translocation was tested using *cyaA* fusions. References: ^1^Marie C, Broughton WJ, Deakin WJ (2001) Rhizobium type III secretion systems: legume charmers or alarmers? Curr Opin Plant Biol 4:336–342; ^2^Lindeberg M et al. (2005) Proposed guidelines for a unified nomenclature and phylogenetic analysis of type III Hop effector proteins in the plant pathogen *Pseudomonas syringae*. Molecular Plant-Microbe Interactions 18:275–282; ^3^Wenzel M, Friedrich L, Göttfert M, Zehner S (2010) The type III-secreted protein NopE1 affects symbiosis and exhibits a calcium-dependent autocleavage activity. Molecular Plant-Microbe Interactions 23:124–129; ^4^Schechter LM, Guenther J, Olcay EA, Jang S, Krishnan HB (2010) Translocation of NopP by *Sinorhizobium fredii* USDA257 into *Vigna unguiculata* root nodules. Appl Environ Microbiol 76:3758–3761; ^5^Marie C et al. (2003) Characterization of Nops, nodulation outer proteins, secreted via the type III secretion system of NGR234. Molecular Plant-Microbe Interactions 16:743–751; ^6^de Lyra MDCCP et al. (2006) Inactivation of the *Sinorhizobium fredii* HH103 rhcJ gene abolishes nodulation outer proteins (Nops) secretion and decreases the symbiotic capacity with soybean. Int Microbiol 9:125–133; ^7^Lorio JC, Kim WS, Krishnan HB (2004) NopB, a soybean cultivar-specificity protein from *Sinorhizobium fredii* USDA257, is a type III secreted protein. Molecular Plant-Microbe Interactions 17:1259–1268; ^8^Hempel J, Zehner S, Göttfert M, Patschkowski T (2009) Analysis of the secretome of the soybean symbiont *Bradyrhizobium japonicum*. Journal of Biotechnology 140:51–58; ^9^Deakin WJ, Marie C, Saad MM, Krishnan HB, Broughton WJ (2005) NopA is associated with cell surface appendages produced by the type III secretion system of *Rhizobium* sp. strain NGR234. Molecular Plant-Microbe Interactions 18:499–507; ^10^Süß C et al. (2006) Identification of genistein-inducible and type III-secreted proteins of *Bradyrhizobium japonicum*. Journal of Biotechnology 126:69–77; ^11^Kambara K et al. (2009) Rhizobia utilize pathogen-like effector proteins during symbiosis. Mol Microbiol 71:92–106; ^12^Rodrigues J et al. (2007) NopM and NopD are rhizobial nodulation outer proteins: identification using LC-MALDI and LC-ESI with a monolithic capillary column. J Proteome Res 6:1029–1037; ^13^Dai W-J, Zeng Y, Xie Z-P, Staehelin C (2008) Symbiosis-promoting and deleterious effects of NopT, a novel type 3 effector of *Rhizobium* sp. strain NGR234. J Bacteriol 190:5101–5110; ^14^Viprey V, Del Greco A, Golinowski W, Broughton WJ, Perret X (1998) Symbiotic implications of type III protein secretion machinery in *Rhizobium*. Mol Microbiol 28:1381–1389; ^15^Krishnan HB et al. (2003) Extracellular proteins involved in soybean cultivar-specific nodulation are associated with pilus-like surface appendages and exported by a type III protein secretion system in *Sinorhizobium fredii* USDA257. Molecular Plant-Microbe Interactions 16:617–625; ^16^Zehner S, Schober G, Wenzel M, Lang K, Göttfert M (2008) Expression of the *Bradyrhizobium japonicum* type III secretion system in legume nodules and analysis of the associated *tts* box promoter. Molecular Plant-Microbe Interactions 21:1087–1093.
(XLSX)
