# Supplementary material for: Mutualistic Co-evolution of Type III Effector Genes in Sinorhizobium fredii and Bradyrhizobium japonicum
Source: PLoS Pathog. 2013 Feb 28;9(2):e1003204. doi: 10.1371/journal.ppat.1003204 (PMC3585131; doi:10.1371/journal.ppat.1003204)
Supplement: Table S1 — Statistics for genome assemblies. *Finished prior to initiation of this study; †32mer and 72mer reads were generated on an Illumina IG or GAII, respectively; ‡number of usable paired end reads; §greater than 100 nt in length; ¶estimates based on sum total of contigs; ∥contigs were ranked ordered according to size and the size (kb) of the smallest contig of those sufficient to represent 50% of the genome size is presented; **number of predicted coding sequences greater than 150 nt in length. (PDF) [file ppat.1003204.s003.pdf]

Table S1. Statistics for genome assemblies.

Strain*	Read length (# channels)[†]	# of PE reads[‡]	# of Contigs[§]	Size (Mb)[¶]	N50 (kb)[‖]	Predicted # CDSs[**]
S. fredii NGR234*	-	-	-	6.9	-	6322
S. fredii USDA207	32mer (2)	22,136,363	291	6.5	27.8	6995
S. fredii USDA257	32mer (2)	27,603,487	384	7.0	26.7	6723
B. japonicum USDA6	32mer (3)	20,995,355	788	8.7	19.6	7960
B. japonicum USDA110*	-	-	-	9.1	-	8317
B. japonicum USDA122	72mer (0.5)	19,430,723	186	8.9	31	8107
B. japonicum USDA123	32mer (3)	27,393,134	815	9.1	21.6	8361
B. japonicum USDA124	32mer (3)	14,219,973	1285	8.9	19.3	7943
